# Perceptions and experiences of Congolese midwives implementing a low-cost battery-operated heart rate meter during newborn resuscitation

**DOI:** 10.3389/fped.2022.943496

**Published:** 2022-09-29

**Authors:** Madeline Thornton, Daniel Ishoso, Adrien Lokangaka, Sara Berkelhamer, Melissa Bauserman, Joar Eilevstjønn, Pooja Iyer, Beena D. Kamath-Rayne, Eric Mafuta, Helge Myklebust, Janna Patterson, Antoinette Tshefu, Carl Bose, Jackie K. Patterson

**Affiliations:** ^1^Department of Pediatrics, University of North Carolina at Chapel Hill, Chapel Hill, NC, United States; ^2^School of Public Health, University of Kinshasa, Kinshasa, Democratic Republic of Congo; ^3^Department of Pediatrics, University of Washington, Seattle, WA, United States; ^4^Strategic Research Department, Laerdal Medical, Stavanger, Norway; ^5^RTI International, Durham, NC, United States; ^6^American Academy of Pediatrics, Itasca, IL, United States

**Keywords:** neonatal resuscitation, Helping Babies Breathe, electronic heart rate monitoring, low-income countries (LMICs), stillbirth

## Abstract

**Background:**

900,000 newborns die from respiratory depression each year; nearly all of these deaths occur in low- and middle-income countries. Deaths from respiratory depression are reduced by evidence-based resuscitation. Electronic heart rate monitoring provides a sensitive indicator of the neonate's status to inform resuscitation care, but is infrequently used in low-resource settings. In a recent trial in the Democratic Republic of the Congo, midwives used a low-cost, battery-operated heart rate meter (NeoBeat) to continuously monitor heart rate during resuscitations. We explored midwives' perceptions of NeoBeat including its utility and barriers and facilitators to use.

**Methods:**

After a 20-month intervention in which midwives from three facilities used NeoBeat during resuscitations, we surveyed midwives and conducted focus group discussions (FGDs) regarding the incorporation of NeoBeat into clinical care. FGDs were conducted in Lingala, the native language, then transcribed and translated from Lingala to French to English. We analyzed data by: (1) coding of transcripts using Nvivo, (2) comparison of codes to identify patterns in the data, and (3) grouping of codes into categories by two independent reviewers, with final categories determined by consensus.

**Results:**

Each midwife from Facility A used NeoBeat on an estimated 373 newborns, while each midwife at facilities B and C used NeoBeat an average 24 and 47 times, respectively. From FGDs with 30 midwives, we identified five main categories of perceptions and experiences regarding the use of NeoBeat: (1) Providers' initial skepticism evolved into pride and a belief that NeoBeat was essential to resuscitation care, (2) Providers viewed NeoBeat as enabling their resuscitation and increasing their capacity, (3) NeoBeat helped providers identify flaccid newborns as liveborn, leading to hope and the perception of saving of lives, (4) Challenges of use of NeoBeat included cleaning, charging, and insufficient quantity of devices, and (5) Providers desired to continue using the device and to expand its use beyond resuscitation and their own facilities.

**Conclusion:**

Midwives perceived that NeoBeat enabled their resuscitation practices, including assisting them in identifying non-breathing newborns as liveborn. Increasing the quantity of devices per facility and developing systems to facilitate cleaning and charging may be critical for scale-up.

## Introduction

Intrapartum-related events result in an estimated two million stillbirths and neonatal deaths annually across the globe (1). The vast majority of these deaths occur in low- and middle-income countries (LMICs) where access to technology to support fetal and postnatal monitoring is limited, thus increasing the burden of perinatal asphyxia (1). Neonatal mortality from intrapartum-related causes is reduced by basic resuscitation practices (2, 3). Basic resuscitation consists of tactile stimulation, clearance of the airway when needed, and bag-mask ventilation (BMV) (4). These basic resuscitation practices are typically taught through standardized resuscitation training programs. One such program is Helping Babies Breathe (HBB), an evidence-based educational program developed by the American Academy of Pediatrics and other global collaborators to teach neonatal resuscitation in resource-limited settings (5). A meta-analysis demonstrated that HBB implementation reduces newborn mortality in the first 24 h after birth by at least 30% (6).

The HBB resuscitation algorithm relies on continuous evaluation of the newborn's respiratory status to determine when to initiate and proceed through basic resuscitation steps. This emphasis on breathing is pragmatic for low-resource settings given the predominance of births attended by a single provider, and the reliance on umbilical cord palpation or auscultation of the chest to evaluate heart rate (HR). Given the difficulty of distinguishing liveborn from stillborn infants with these methods of HR evaluation, HBB recommends initiation of resuscitation for all non-macerated neonates regardless of HR detection. HBB also relies on an initial evaluation of chest rise to determine the effectiveness of BMV, recommending HR assessment after 1 min of BMV with good chest rise or sooner in the algorithm if there is a second skilled birth attendant (SBA) present. In contrast, resuscitation algorithms in high-income countries (HICs), such as the Neonatal Resuscitation Program and Neonatal Life Support, recommend continuous evaluation of HR with electronic monitoring to guide resuscitation during positive pressure ventilation (7, 8). This practice is in keeping with the International Liaison Committee on Resuscitation's recommendation for use of electrocardiogram (ECG) to provide a rapid and accurate assessment of HR in newborns requiring resuscitation at birth (9). Although electronic HR monitoring during newborn resuscitation has become standard practice in HICs, its relatively high cost limits its uptake for newborn resuscitation in LMICs (10).

In a recent clinical trial in the Democratic Republic of the Congo (DRC), HBB-trained midwives integrated a low-cost, battery-operated HR meter (NeoBeat™; Laerdal Global Health, Stavanger, Norway) into their resuscitation practice for 20 months (11). During the trial, midwives used the newborn HR displayed by NeoBeat to assist them in classifying flaccid infants as liveborn or stillborn, and to guide their initiation of BMV and corrective steps. They also developed systems for charging and cleaning the device between patients. To understand issues relevant to scale-up of NeoBeat for HR monitoring after birth in LMICs, we investigated the midwives' perceptions and experiences regarding their use of NeoBeat during the clinical trial.

## Methods

### Study design

We used a qualitative study design involving focus group discussions (FGDs) to explore midwives' perceptions and experiences regarding the incorporation of NeoBeat into their resuscitation practices.

### Setting

Our clinical trial of electronic HR monitoring during newborn resuscitation took place in three health facilities in Kinshasa, DRC (11). The maternity wards at the participating facilities supported ~1,051 to 4,248 deliveries each year during the trial. Facilities A and C were Catholic, and Facility B was public; only Facility B offered Cesarean section. Midwives provided basic newborn resuscitation including BMV at all three facilities as standard of care. Electronic HR monitoring was not available in the study hospitals prior to the trial.

### NeoBeat HR meter

During the 20-month intervention phase of our clinical trial, HBB-trained midwives implemented electronic HR monitoring during newborn resuscitation using NeoBeat. NeoBeat is a CE marked, battery-operated C-shaped device that digitally displays a newborn's HR using dry-electrode ECG (12). It is affordable for low-resource settings, rechargeable, and can be placed rapidly on a newborn by a single provider with display of HR within 10–13 s of placement (https://shop.laerdalglobalhealth.com/product/neobeat/) (13–15).

Each health facility received three NeoBeat devices to use during the trial. All midwives received formal training on NeoBeat from research staff, including interpretation of its digital output, placement on newborns, and maintenance of the device (cleaning, charging, storage) (11). This training was embedded into HBB training and added ~2 h. Following training, each facility developed a system for cleaning and charging the device between patients. A study physician was in contact with the facilities on a weekly basis, and available to troubleshoot any technical difficulties with the device. A study nurse was also present for at least 40 h per week at each facility to conduct observations of resuscitations.

During the trial, midwives placed NeoBeat on all non-breathing newborns at birth and used HR to guide their classification of flaccid infants as liveborn or stillborn. During the second half of the intervention phase (08/17/19-06/22/20), midwives also used HR to guide their initiation of BMV and corrective steps for ventilation. Facility A participated in a secondary study that used NeoBeat in breathing newborns at birth; at this facility, midwives placed NeoBeat on all newborns at the time of birth.

### Data collection

We conducted FGDs in July 2020, ~20 months after midwives began using NeoBeat. Two DRC-based study staff facilitated one FGD at each of the three study facilities with a convenience sample of 10 experienced midwives per facility. During audio-recorded discussions, facilitators solicited midwives' perceptions of and experience with NeoBeat through open-ended and neutral questions using an FGD guide (Appendix 1). Every midwife included in the FGD had the opportunity to respond to each question if desired during the hour-long conversation. All discussions occurred in Lingala, a Bantu language spoken in the DRC. DRC-based study staff transcribed all audio-recorded FGDs and translated the transcriptions from Lingala to French. A US-based study team member (MT) with fluency in both English and French translated the French transcript into English.

### Data analysis

Two reviewers (MT and Jackie Patterson) analyzed data using the qualitative content analysis method (16). Together, both reviewers coded English transcripts using Nvivo 12 Pro software (Alfasoft, Göteborg, Sweden) and subsequently analyzed codes to identify patterns in the data. Next, the reviewers independently grouped codes into categories and then determined final categories by consensus. Table 1 demonstrates the process of how FGD transcriptions were coded and categorized.

**Table 1:** Illustration of qualitative content analysis with examples of transcribed text, assigned code and category.

**Transcribed text**	**Code**	**Category**
*NeoBeat loses battery after 3 or 4 deliveries but often around two hours*	Device battery life	Challenges of use of NeoBeat included cleaning, charging and insufficient quantity of devices.
*We are happy to use it and continue since it has strengthened our capacities*	Future application	Providers desired to continue using NeoBeat and to expand its use beyond resuscitation and their own facilities.

## Results

### Estimated frequency of use of NeoBeat

In the clinical trial, midwives used NeoBeat in 96% of observed deliveries and 26% of newborns were apneic or not breathing well by 30 s after birth. In facility A where midwives used NeoBeat for all newborns at birth, each midwife used NeoBeat on an estimated 373 newborns over the course of the trial (assuming a normal distribution of cases; Table 2). In facilities B and C where midwives used NeoBeat for non-breathing newborns at birth, each midwife used NeoBeat on 24–47 newborns over the course of the trial (assuming a normal distribution of cases).

**Table 2:** Estimated frequency of use of NeoBeat per midwife.

**Facility**	**Number of births during the study period**	**Number of midwives**	**Estimated births for which NeoBeat was applied**	**Estimated cases of NeoBeat use per midwife^a^**
A	5,822	15	5,589^b^	373
B	1,536	16	383^c^	24
C	3,779	20	943^c^	47

a Estimated cases assume a normal distribution between providers.

b Calculated using number of births × 96% rate of NeoBeat use.

c Calculated using number of births × 96% rate of NeoBeat use × 26% non-breathing newborns since these facilities only applied NeoBeat for non-breathing newborns.

### Perceptions and experiences of midwives using NeoBeat

We identified five main categories of perceptions and experiences regarding the use of NeoBeat from FGDs.

#### Providers' initial skepticism evolved into pride and a belief that NeoBeat was essential to resuscitation care

Midwives were previously accustomed to assessing newborn HR *via* palpation of the umbilical cord or auscultation of the chest. When reflecting on the first few weeks of using NeoBeat including initial reactions to the technology, a common theme was a concern that NeoBeat increased workload:

*At first, it was a heavy load. It was seen as an extra burden [of work] on top of what we were already doing. (Facility B)*

Other initial impressions included a belief that NeoBeat was a “nuisance.” Several midwives expressed initial fear evolving into pride:

*I was scared but when it arrived, I used it [and] I was proud of its use. (Facility A)*

As midwives began incorporating NeoBeat into their daily practice, they became increasingly comfortable with the device. After extensive use, midwives expressed the belief that NeoBeat is both helpful and essential to the resuscitation of newborns:

…*we started to use it and with experience we have noticed that the device is very important and helps us in the resuscitation of children and we really liked it, but at the beginning we thought it was a burden but then we really understood that it was necessary. (Facility B)*

*It is part of essential care that is an obligation to give to all babies. (Facility A)*

#### Providers viewed NeoBeat as enabling more effective resuscitation and increasing their capacity

Heart rate is a sensitive indicator of both the need for resuscitation as well as the response to interventions. In LMICs, HR is infrequently evaluated during newborn resuscitation due to the reliance on umbilical cord palpation and auscultation of the chest to detect HR. With the incorporation of NeoBeat into resuscitation practice, midwives reported that the HR from NeoBeat allowed them to quickly identify newborns who would benefit from resuscitation. They also noted that using NeoBeat allowed them to carry out the steps of resuscitation in a more efficient and easier manner:

*As soon as you place it directly in the first minutes you know if this baby needs to be resuscitated or not and even during the resuscitation, I no longer have…to search for what I must do or what state the baby is in so directly I see what I'm doing, it helps positively. (Facility A)*

*In the time when the baby is born and we notice that he is in distress and we confirm that this baby [is] really appearing dead everyone confirms it, but since NeoBeat we know to say that this baby has a problem of resuscitation and he is asphyxiated in the instant that we put NeoBeat [on] and it works…the movements become fast…The use of the NeoBeat makes the task easier. (Facility B)*

Many midwives stated that using NeoBeat facilitated resuscitation and allowed them to treat depressed newborns more effectively:

*Today if a child is born without hope of life and we place it, and if the numbers come out, we are reassured and we start the resuscitation maneuvers, we say that it has increased or it falls and we search by all means the techniques to save that child according to NeoBeat. (Facility C)*

In addition, several midwives discussed NeoBeat's role in capacity building, sharing the belief that the new technology for resuscitation improved care:

*When we heard that the new technology came, we understood that the science is evolving, it is welcome to strength[en] the capacities of providers in the field to better save lives in institutions of health; it was welcome[d] for capacity building. (Facility B)*

#### NeoBeat helped providers identify flaccid newborns as liveborn, leading to hope and the perception of saving of lives

Midwives reflected on whether NeoBeat changed the way they resuscitate newborns, including specifically how NeoBeat helped or hindered care for babies who are not breathing at birth. Several midwives perceived that NeoBeat saved the lives of newborns that may have been misclassified as stillborn without the device:

*Yes if NeoBeat was not there the baby should be declared stillborn since we did not hear a heartbeat even in the umbilical nor by auscultation we should declare it stillborn. (Facility C)*

One midwife shared a specific instance in which she believed that a flaccid newborn was stillborn and was surprised to see a heartbeat when using NeoBeat:

*What I say is that I saw it myself, during auscultation we did not hear the fetal heartbeat and when the baby came out flaccid and I myself judged him stillborn and after a time I told myself to place first NeoBeat, we noticed there was a heartbeat and nothing in the umbilical cord and right away we started the resuscitation of that baby and he was saved and left for home. (Facility C)*

Another midwife reflected on her clinical practice before NeoBeat, sharing a story in which she noticed a newborn breathing that had been classified as stillborn and received no medical care. In this exemplary quote, she imagines how this experience may have been different with NeoBeat, emphasizing the role of electronic HR monitoring in identifying flaccid newborns as stillborn or liveborn:

*One time I found a baby already wrapped up and I asked the midwives how did you leave this child, they said that it is a stillborn and I said to them go see him he is breathing and they said but he was already dead no and that was before NeoBeat and if NeoBeat had been there they should not have been surprised like that, so it is to say that NeoBeat helped since at the beginning when we had an apparent stillborn we concluded that it is a stillbirth, but with NeoBeat there is still a heartbeat, it still gives us the opportunity to resuscitate and to bring this baby back to life hence that has greatly changed the care of newborns in our maternity ward. (Facility B)*

One midwife shared that the success of resuscitation using NeoBeat motivated her to continue using the device:

*The success of the resuscitation helped us a lot. During resuscitation, sometimes we were faced with a child who appeared to be dead, but with the use of the NeoBeat we realized that he was not dead. Once resuscitated, the child survives. This encourages us. (Facility B)*

#### Challenges of use of NeoBeat included cleaning, charging and insufficient quantity of devices

Midwives reflected on the biggest barriers to using NeoBeat during newborn resuscitations, identifying three main challenges of use: cleaning, charging and insufficient quantity of devices.

Proper cleaning and disinfection of NeoBeat requires several steps: (1) removing all visible soil with a soapy cloth, (2) wiping clean with a cloth dampened with water, (3) drying, (4) wiping with 70% ethanol, (5) spraying with 70% ethanol and repeating to ensure NeoBeat remains wet for 10 min, (6) wiping with a cloth dampened with water, and (7) drying. Common themes for barriers to keeping NeoBeat clean included insufficient materials for cleaning (in particular, gloves and ethanol), insufficient time to clean between patients and NeoBeat left dirty between uses. One midwife described truncating the cleaning process when NeoBeat was needed for another patient:

…*NeoBeat had to remain in the ethanol solution for 10 minutes. However, after the material were put back in order and the NeoBeat had not yet been in the ethanol for 10 minutes, it was sometimes used for other deliveries. Thus, the disinfection time was not respected. (Facility A)*

Inadequate battery life and the associated need for intermittent charging was another commonly discussed barrier to using NeoBeat during newborn resuscitations. One midwife noted:

*NeoBeat loses battery after 3 or 4 deliveries but often around two hours. (Facility A)*

Another common barrier to using NeoBeat cited among midwives across health facilities was an insufficient quantity of devices for the need. As described in the exemplary quote below, midwives noted that often when the NeoBeat device was being cleaned, a delivery would require the use of NeoBeat and there would not be a device available for use:

*Insufficient quantity of NeoBeats, the little that we have could be in sterilization and we wait or there is a birth and we need NeoBeat, if we had a sufficient quantity it would facilitate the work by exchanging the NeoBeat rather than wait for the one that is in sterilization, soon we must be given the sufficient quantity to facilitate the exchange; we do not wish for a child to be born always suffering but to have only enough. (Facility C)*

As a result of this barrier to use, many midwives specifically asked to increase the number of NeoBeat devices at their facility.

#### Providers desired to continue using NeoBeat and to expand its use beyond resuscitation and their own facilities

Midwives unanimously expressed a desire to continue to use NeoBeat in their clinical practice beyond the clinical trial. As of December 2021, midwives were electively using NeoBeat as part of their resuscitation practice at all three facilities.

Midwives at Facilities B and C were asked whether they believed NeoBeat should be expanded to babies breathing well at birth. Some midwives expressed a desire to expand to babies breathing well at birth, while others did not. As expressed in the following exemplary quote, midwives who were not in favor of expanding the use of NeoBeat to babies breathing well at birth reasoned that when a baby is crying after delivery, it is clearly not stillborn and does not require resuscitation:

*I would say no since NeoBeat is only intended for the recovery of the dead, the babies who we consider really dead and if we place NeoBeat and it shows us something to get them back, but the babies who are born normally with normal cries and a good heartbeat I do not see the importance of using NeoBeat…that is why I say that to place it on all babies is a burden. (Facility B)*

Some midwives expressed an interest in using NeoBeat in other care beyond the delivery room. One midwife described the ways in which she believed that NeoBeat would be helpful in all babies, including in postnatal monitoring:

*If we have a permanent supply of NeoBeat…, we will use it in all children without distinction. It will help us monitor the condition of other babies. We can see that a baby is born without any problems, but a few hours later or even a few days later he or she has breathing problems if he or she is still in the maternity ward. Even before referring a child to another health facility, the NeoBeat allows you to determine the heart rate quickly instead of using a stethoscope. If we have a large supply, we can use the NeoBeat on all children. We can use it during postpartum monitoring. (Facility A)*

A final common theme mentioned by the midwives was their desire to expand use of NeoBeat to other facilities in the DRC:

*We ask to extend it in other hospitals to save lives. (Facility A)*

*We must make an effort so that it arrives [to] everyone in the DRC. (Facility A)*

## Discussion

In this qualitative study, we explored midwives' perceptions and experiences regarding the use of a battery-operated HR meter called NeoBeat for newborn resuscitation during a clinical trial in the DRC. Although midwives expressed initial skepticism regarding use of NeoBeat, most midwives came to believe that NeoBeat was essential to resuscitation care, enabling more effective resuscitation of newborns, helping to identify flaccid newborns as liveborn, and increasing their capacity. While the midwives had a positive impression of NeoBeat and unanimously expressed a desire to continue using the device after the trial ended, they shared several barriers to its use including charging and cleaning.

Continuous electronic HR monitoring may be an important strategy to ensure initiation of resuscitation for flaccid newborns. Recognizing the challenge of distinguishing liveborn from stillborn infants, HBB recommends initiation of resuscitation for all non-macerated newborns with continuation for at least 10 min. The experiences of these Congolese midwives suggests that HBB-trained providers may rapidly conclude an infant is stillborn in the absence of electronic HR monitoring, and thereafter deviate from the recommendation to attempt resuscitation. In our study, there were several accounts of newborns that appeared stillborn who were accurately identified as liveborn based on the HR provided by NeoBeat. The HR displayed by NeoBeat led midwives to initiate resuscitation in cases they may have previously presumed futile. Our findings indicate that continuous electronic HR monitoring changed the tendency to rapidly conclude flaccid neonates are stillborn.

Midwives reported positive experiences with NeoBeat that led to the perception that NeoBeat saved lives. In several cases of resuscitating flaccid neonates, midwives described the newborn “coming back to life” with basic resuscitation. Despite these positive experiences, the clinical trial did not demonstrate an overall reduction in perinatal death (11). Although our qualitative study indicates that electronic HR monitoring may prompt initiation of resuscitation for flaccid newborns, initiation of resuscitation does not necessarily reduce mortality. Furthermore, while midwives in our study reported improved clinical ascertainment of stillbirth due to electronic HR monitoring, 20 percent of newborns with HR monitoring who were classified by providers as stillborn in this trial had a documented HR and were thus determined to be misclassified (11). When initiation of resuscitation of a flaccid infant still results in perinatal death, factors beyond objective, clinical data such as cultural influences and concerns for culpability may be stronger motivators in an SBA's classification of the death as fetal vs neonatal.

Midwives identified several barriers to using NeoBeat linked to its reprocessing for the next newborn. The need to clean and charge NeoBeat sometimes led to unavailability of the device for subsequent births, or use of the device before cleaning had been completed. The materials required to complete the recommended disinfection process were also noted to be challenging to acquire; in the DRC, denatured alcohol is less costly and more frequently stocked in hospitals as compared to 70% ethanol. As such, following the clinical trial, facilities electing to continue using NeoBeat transitioned to cleaning it with denatured alcohol per what was locally feasible. Our findings are consistent with prior literature on the reprocessing of basic neonatal resuscitation equipment in LMICs. The premature removal of resuscitation equipment from cleaning processes due to urgent need to use in a subsequent neonatal resuscitation has been previously reported (17). In 2016, PATH, a non-governmental organization of global health innovators, convened an international group of stakeholders to develop guidelines for the reprocessing of neonatal resuscitation equipment in resource-constrained settings (18). These guidelines were incorporated into HBB training in the program's second edition; however, there has been on-going concern that these recommendations may be too challenging to implement due to the resources required (17). A qualitative study exploring barriers and facilitators to disinfection of neonatal resuscitation equipment with midwives in Kenya called for partnership with front-line providers to revise cleaning guidelines to promote effective, feasible cleaning processes (19). In light of our findings and prior literature on reprocessing, we suggest that implementation of NeoBeat should be accompanied by systems for cleaning that include a steady supply-chain of cleaning products and clearly defined roles and responsibilities for cleaning. Additional research to determine safety of cleaning with more accessible cleaning solutions may enhance scale-up. Charging was also noted by midwives as a barrier to use of NeoBeat. To address this barrier, implementation of NeoBeat should include the development of a system for charging with regular monitoring of the device battery-life and intermittent charging. Increasing the quantity of devices available may mitigate the reprocessing burden associated with cleaning and charging the device.

Midwives expressed growing comfort and appreciation for NeoBeat with use over time. These findings are similar to the experiences of Tanzanian SBAs with an electronic fetal HR monitor called Moyo (Developer: Laerdal Global Health; Stavanger, Norway) (20). This monitor was evaluated in a facility that used intermittent auscultation of fetal heart tones with a Pinard device as standard of care. Investigators used FGDs to evaluate acceptability of the device among SBAs. As in our study, SBAs reported initial skepticism followed by growing trust in the Moyo device after gaining confidence in its use. Another qualitative study investigating the feasibility, usability, and acceptability of pediatric lung ultrasound among healthcare providers in Pakistan and Mozambique identified caregiver acceptance as vital to feasibility of technology uptake and success in a clinical setting (21). Healthcare providers expressed growing acceptance of ultrasound over time with increased use in clinical practice. These studies suggest that practice with devices in the clinical setting over time may be key to the acceptability of new technologies.

Our study is strengthened by the long timeframe of 19 months of implementation of NeoBeat in a clinical setting prior to this qualitative evaluation. We assessed midwives' experiences and perceptions in FGDs, allowing for interaction between midwife participants and facilitators in a semi-structured setting. FGDs were conducted in Lingala, the midwives' native language. FGDs were led by study personnel who spoke Lingala fluently and are trusted members of the community. Limitations of this study include the possibility that midwives may have felt pressured to speak favorably about their experience with NeoBeat given the use of study personnel as facilitators. Furthermore, there is also the risk of group effect bias in FGDs in which participants agree with each other's opinions to uphold a consensus. It is possible that participants with opinions differing from the majority may not have felt comfortable voicing these contradictory beliefs. Although midwives reported increasing comfort and appreciation of NeoBeat over time, there is also a potential risk of recall bias as we did not collect qualitative data on midwives' perceptions and experiences of using NeoBeat during the early stages of the trial. We analyzed data in English rather than the original language in which the FGDs were conducted, and thus cannot exclude the possibility that meaning may have been lost in the translation process. Finally, the perceptions and experiences of the midwives using NeoBeat reflect implementation of NeoBeat within a clinical trial. As the clinical trial included instruction in the use of HR to enable accurate classification of liveborn vs stillborn infants as well as HR-guided resuscitation, their perceptions and experiences may not be generalizable to those implementing the device without the addition of this clinical training.

## Conclusion

Midwives incorporating a battery-operated HR meter called NeoBeat into their newborn resuscitation practice in the DRC perceived that continuous electronic HR monitoring enabled their resuscitation. These midwives reported initiating resuscitation of newborns they may have previously presumed stillborn, leading to a change in their tendency to rapidly conclude flaccid neonates as stillborn due to the use of NeoBeat. Health facilities seeking to adopt NeoBeat should clearly define systems for cleaning and charging the device as well as ensure a sufficient quantity of devices in order to facilitate its uptake.

## Data availability statement

The original contributions presented in the study are included in the article/Supplementary material, further inquiries can be directed to the corresponding author.

## Ethics statement

The studies involving human participants were reviewed and approved by University of North Carolina IRB. The patients/participants provided their written informed consent to participate in this study.

## Author contributions

MT, CB, and JKP: study conception and design and draft manuscript preparation. DI and AL: data collection. MT, PI, and JKP: analysis and interpretation of results. All authors reviewed the results and approved the final version of the manuscript.

## Funding

This study was funded by Doris Duke Charitable Foundation Grant #2020143, Saving Lives at Birth Grand Challenge Award, Thrasher Early Career Award, Laerdal Foundation Award, and The Frederick S. Neuer, MD '71 Medical Student Travel Endowment Fund.

## Conflict of interest

Authors JE and HM were both employed by Laerdal Global Health (LGH), the company that developed NeoBeat. The remaining authors declare that the research was conducted in the absence of any commercial or financial relationships that could be construed as a potential conflict of interest.

## Publisher's note

All claims expressed in this article are solely those of the authors and do not necessarily represent those of their affiliated organizations, or those of the publisher, the editors and the reviewers. Any product that may be evaluated in this article, or claim that may be made by its manufacturer, is not guaranteed or endorsed by the publisher.
