# Supplementary material for: Perceptions and experiences of Congolese midwives implementing a low-cost battery-operated heart rate meter during newborn resuscitation
Source: Front Pediatr. 2022 Sep 29;10:943496. doi: 10.3389/fped.2022.943496 (PMC9557145; doi:10.3389/fped.2022.943496)
Supplement: Supplementary file 1 [file Table_1.DOCX]

**Appendix 1: Focus Group Discussion Guide, English Version**

| **Introduction** |
| --- |
| Hello. My name is **[name]**. Thank you for taking time to participate today. We’ll be here for about an hour. The reason we’re here today is to gather your opinions and attitudes about your experience with NeoBeat. I’m going to lead our discussion today. I will be asking you questions and then encouraging and moderating our discussion. I also would like you to know this focus group will be audio recorded. The identities of all participants will remain confidential. The recording allows us to review our discussion for the purposes of improving NeoBeat and reporting on this study.    **Rules for Group:**  To make our conversation easier, I’d like to go over some rules.   1. Only one person speaks at a time. This is important as our goal is to make a written transcript of our conversation today. It is difficult to capture conversation on our audio recording if there are multiple voices at once. 2. Please avoid side conversations. 3. Everyone doesn’t have to answer every single question, but I’d like to hear from each of you at some point today. 4. This is a confidential discussion. I will not report your names or who said what to your colleagues or supervisors. Names of participants will not be included in the final report about this meeting. 5. There are no “wrong answers,” just different opinions. Say what is true for you, even if you’re the only one who feels that way. Don’t let the group sway you. But if you do change your mind, let me know.     Are there any questions?    **Introduction of Participants:** Now I’d like to go around the table. Each person should state their first name only and tell me in a few words about something they did this week that was important or fun. Again, no last names or other information that would identify you and keep it short, please. |
| **Focus Group Questions**    *Think back to the first few weeks you used NeoBeat during newborn resuscitations.*   1. What were your initial reactions to the technology? 2. How did you feel about incorporating NeoBeat into your clinical practice? 3. What were some challenges to using NeoBeat? How did these challenges evolve over time?     *The following questions are about the NeoBeat device itself.*   1. Describe any problems you have had using the NeoBeat device? (Probing: e.g., difficulty turning it on, reading the display, placing it on the baby, charging it etc.) 2. Have you noticed any skin injury or rash on the baby after using NeoBeat? 3. What has made keeping NeoBeat clean difficult? 4. What has helped your clinical team keep NeoBeat clean? 5. What do you do, if anything, to confirm that NeoBeat is clean?   *Consider if NeoBeat has altered the care you provide to newborns.*   1. Has NeoBeat changed the way you resuscitate newborns? 2. How does NeoBeat help or hinder care for babies who are not breathing at birth? 3. Facility A follow-up question: How has NeoBeat changed care of babies who ***are*** breathing at birth? 4. Facilities B and C follow-up question: If you used NeoBeat in all babies, would NeoBeat change the care of babies who ***are*** breathing at birth? 5. Has NeoBeat altered the care you provide to flaccid newborns? If yes, how? 6. Has NeoBeat changed how you identify stillborn infants? If yes, how? 7. Has NeoBeat altered the care you provide to stillborn infants? If yes, how? 8. What does heart rate tell you about the newborn’s health?     *Using technology in medical practice can be both burdensome and valuable.*   1. Describe your experience, both negative and positive, using NeoBeat in clinical practice. (Probing: e.g. how is using NeoBeat burdensome? How is using NeoBeat valuable to you?) 2. Has using NeoBeat interfered with or delayed resuscitation? Please explain. 3. What are the biggest barriers to using NeoBeat during newborn resuscitations? (Probing: e.g., it delays the resuscitation process, cleaning is time-consuming, etc.) 4. Tell me about your impression of mothers’ opinions of NeoBeat. (Probing: e.g., did mothers seem to like it or not like it? Why do you think they liked it or didn’t like it?)     *Some of you may not be interested in continuing to use NeoBeat after the study has finished, and others may be interested.*   1. Tell me how you feel about the possibility of continuing to use NeoBeat at your hospital. 2. For those of you who would ***not*** like to continue using NeoBeat, tell me some reasons why. 3. For those of you who would like to continue using NeoBeat, tell me some reasons why.     *If your hospital decides to continue to use NeoBeat after the study ends, consider how you would incorporate the device into your care of newborns.*   1. What would you change about how you are currently using NeoBeat? 2. Would you use NeoBeat for every newborn? Only for newborns who are not breathing? 3. In what circumstances would you not want to use NeoBeat? (Probing: e.g., certain times of day? certain staffing conditions?)      1. Is there anything else you would like to tell me about your experience with NeoBeat? |
